# Supplementary material for: Optimal Nozzle Position and Patient’s Posture to Enhance Drug Delivery into the Peritoneum during Rotational Intraperitoneal Pressurized Aerosol Chemotherapy in a Swine Model
Source: J Pers Med. 2022 Oct 31;12(11):1799. doi: 10.3390/jpm12111799 (PMC9695236; doi:10.3390/jpm12111799)
Supplement: Supplementary file 1 [file jpm-12-01799-s001.zip › jpm-1940629-supplementary.pdf]

Supplementary Document S1.

Members of KoRIA trial group

Eun Ji Lee (Department of Obstetrics and Gynecology, Chung-Ang University Hospital, Seoul 06973, Korea)

Ga Won Yim (Department of Obstetrics and Gynecology, Dongguk University College of Medicine, Goyang 10326, Korea)

Hee Seung Kim (Department of Obstetrics and Gynecology, Seoul National University College of Medicine, Seoul 03080, Korea)

Ji Won Park (Department of Surgery, Seoul National University Hospital, Seoul 03080, Korea)

Kyung Ah Pak (Agaon Fertility Clinic, Seoul 08391, Korea)

San-Hui Lee (Department of Obstetrics and Gynecology, Wonju Severance Christian Hospital, Yonsei University College of Medicine, Wonju 26426, Korea)

Seung-Hyuk Shim (Department of Obstetrics and Gynecology, Research Institute of Medical Science, Konkuk University School of Medicine, Seoul 05030, Korea)

Seungmee Lee (Department of Obstetrics and Gynecology, Keimyung University School of Medicine, Daegu 42601, Korea)

Soo Hyun Oh (Department of Obstetrics and Gynecology, Gil Medical Center, Gacheon University College of Medicine, Incheon 21565, Korea)

Soo Jin Park (Department of Obstetrics and Gynecology, Seoul National University Hospital, Seoul 03080, Korea)

Suk-Joon Chang (Division of Gynecologic Oncology, Department of Obstetrics and Gynecology, Ajou University School of Medicine, Suwon 16499, Korea)

Sung Jong Lee (Department of Obstetrics and Gynecology, Seoul St. Mary’s Hospital, College of Medicine, The Catholic University of Korea, Seoul 06591, Korea)
